# Combination of paeoniflorin and calycosin-7-glucoside alleviates ischaemic stroke injury via the PI3K/AKT signalling pathway

**DOI:** 10.1080/13880209.2022.2102656

**Published:** 2022-08-07

**Authors:** Peng-Cheng Wang, Sheng-Xin Wang, Xiang-Li Yan, Ying-Ying He, Min-Chun Wang, Hao-Zhen Zheng, Xu-Guang Shi, Yong-Heng Tan, Li-Sheng Wang

**Affiliations:** College of Chinese Materia Medica, Guangzhou University of Chinese Medicine, Guangdong, China

**Keywords:** Ischaemia/reperfusion injury, HT22 cells, neuroprotective effects, OGD/R injury, synergistic enhancement, synergistic interaction

## Abstract

**Context:**

Paeoniflorin (PF) and calycosin-7-glucoside (CG, *Paeonia lactiflora* Pall. extract) have demonstrated protective effects in ischaemic stroke.

**Objective:**

To investigate the synergistic effects of PF + CG on ischaemia/reperfusion injury *in vivo* and *in vitro*.

**Materials and methods:**

Male Sprague-Dawley rats were subjected to the middle cerebral artery occlusion/reperfusion (MCAO/R). After MCAO/R for 24 h, rats were randomly subdivided into 5 groups: sham, model (MCAO/R), study treatment (PF + CG, 40 + 20 mg/kg), LY294002 (20 mg/kg), and study treatment + LY294002. Males were given via intragastric administration; the duration of the *in vivo* experiment was 8 days. Neurologic deficits, cerebral infarction, brain edoema, and protein levels were assessed *in vivo*. Hippocampal neurons (HT22) were refreshed with glucose-free DMEM and placed in an anaerobic chamber for 8 h. Subsequently, HT22 cells were reoxygenated in a 37 °C incubator with 5% CO_2_ for 6 h. SOD, MDA, ROS, LDH and protein levels were measured *in vitro*.

**Results:**

PF + CG significantly reduced neurobehavioral outcomes (21%), cerebral infarct volume (44%), brain edoema (1.6%) compared with the MCAO/R group. Moreover, PF + CG increased p-PI3K/PI3K (4.69%, 7.4%), p-AKT/AKT (6.25%, 60.6%) and Bcl-2/BAX (33%, 49%) expression *in vivo* and *in vitro*, and reduced GSK-3β (10.5%, 9.6%) expression. *In vitro*, PF + CG suppressed apoptosis in HT22 cells and decreased ROS and MDA levels (20%, 50%, respectively).

**Conclusions:**

PF + CG showed a synergistic protective effect against ischaemic brain injury, potentially being a future treatment for ischaemic stroke.

## Introduction

Ischaemic stroke (IS) is characterized by high morbidity and mortality, with a heavy burden on families and society (De Giuli et al. 2019; Zheng et al. 2019). Due to a narrow therapeutic window and a poor efficacy to recanalize occlusions of large arteries, new therapies for IS are currently being investigated.

Different pathophysiological processes are involved in IS, including the phosphatidylinositol 3-kinase (PI3K)/protein kinase B (AKT) pathway (Zhang et al. 2007; Jie et al. 2015; Peng et al. 2019). Activation of the PI3K/AKT signalling pathway inhibits a number of apoptotic mechanisms and promotes cell cycle progression, thereby promoting cell survival and proliferation (Cao et al. 2019). Moreover, the activated PI3K/AKT pathway significantly reduces brain damage, protecting hippocampal and cortical neurons against hypoxia/reoxygenation-induced apoptosis (Gao et al. 2018; Chen et al. 2019; Li et al. 2019). LY294002 is widely used in the characterization of the phosphatidylinositol kinase signalling pathway, being able to penetrate cells and specifically inhibit PI3K (Wang et al. 2017).

Buyang Huanwu Decoction (BHD), a classical prescription in traditional Chinese medicine (Hsu et al. 2019), created by Qingren Wang in Qing Dynasty, mainly records the theory ‘YIQIHUOXUE’. Previous experiments using *in vivo* microdialysis determined that the paeoniflorin (PF) and calycosin-7-glucoside (CG) are the main components of the affinity components of ‘YIQI and HUOXUE’ in Buyang Huanwu Decoction (Shen et al. 2019a, 2019b). PF is a major constituent contained in paeony root that inhibits prothrombin synthesis and platelet aggregation, dilates peripheral arterioles and improves blood microcirculation (Zhang et al. 2015, 2017; She et al. 2019). CG has demonstrated the ability to dilate blood vessels and protect the blood-brain barrier in experimental cerebral ischaemia (Fu et al. 2014). Together, PF and CG may play a role against brain damage following cerebral ischaemia. However, potential mechanisms underlying their beneficial effects are still unknown. In this study, we investigated the signalling pathways involved in the neuroprotective effects of PF combined with CG *in vitro* and *in vivo*.

## Materials and methods

### Chemicals and reagents

**Figure 1.**
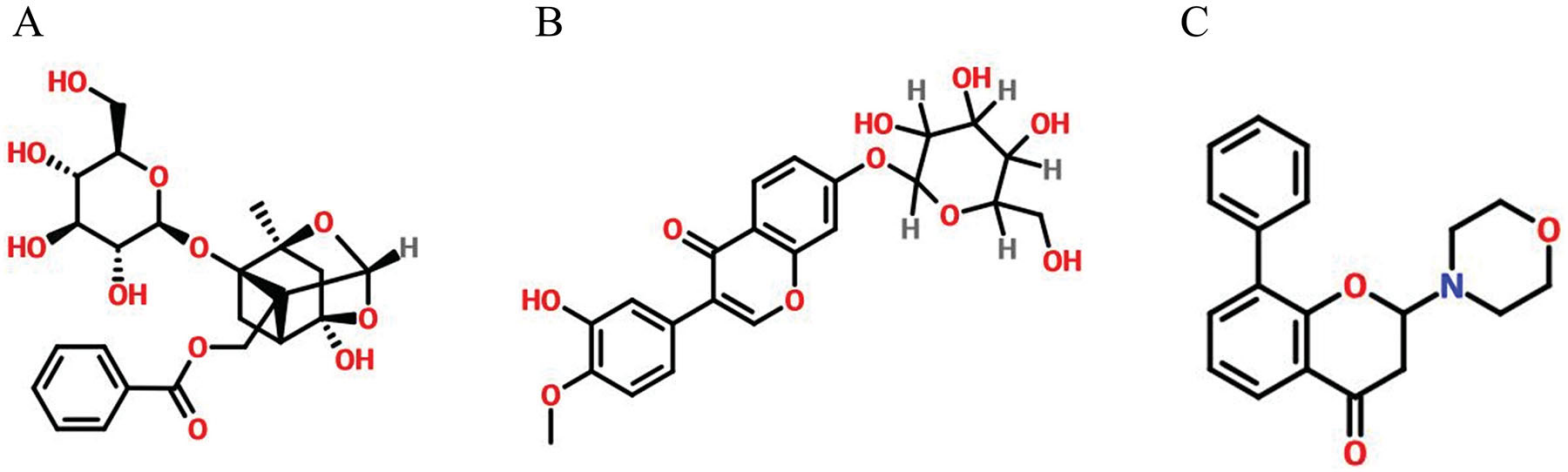
Chemical structure of (A) paeoniflorin (B) calycosin-7-glucoside and (C) LY294002.

PF (Figure 1(A), purity ≥ 98%) and CG (Figure 1(B), purity ≥ 98%) were purchased from Chroma Pharmaceuticals, Inc. (Austria). LY294002 (Figure 1(C), purity ≥ 98%) was purchased from SCN Pharmaceuticals, Inc. Annexin V-FITC/PI Apoptosis and JC-10 Apoptosis detection kits were purchased from Jiangsu Kaiji Biological Technology Co., Ltd. Superoxide dismutase (SOD) assay kit, malondialdehyde (MDA) assay kit, total reactive oxygen species (ROS) assay kit, and lactate dehydrogenase (LDH) assay kit (Cat: 20190416) were provided by Nanjing Jiancheng Bioengineering Institute. SybrGeen qPCR Mastermix and qPCR RT Kit were purchased from DBI Bioscience. Anti-PI3K, anti-AKT, anti-p-PI3K, anti-p-AKT, anti-BAX, anti-Bcl-2, anti-GSK-3β, anti-β-actin and goat anti-rabbit IgG (H + L) antibodies were obtained from Jiangsu Kinke Biological Research Centre Co., Ltd. (Jiangsu, China) (Shang et al. 2018; Li et al. 2020; Wu et al. 2020).

#### Middle cerebral artery occlusion/reperfusion (MCAO/R) rat model

Sixty adult male Sprague-Dawley rats, weighing 285 ± 15 g, were provided by Hunan SJA Laboratory Animal Co., Ltd. (Hunan, China). All experimental procedures were approved by the Animal Ethics Committee of Guangzhou University of Chinese Medicine (approval no. ZYD-2021-182) and performed according to the guidelines of the European Community and the National Institutes of Health (USA).

The middle cerebral artery occlusion/reperfusion (MCAO/R) model was implemented according to previous experiments (Wang et al. 2013). The rats were anaesthetized with urethane (intraperitoneal injection, 1.2 g/kg) and placed on a heating pad set at 37 °C. A midline neck incision was made to carefully isolate the right common carotid artery, the external carotid artery and the internal carotid artery. Common and external carotid arteries were sutured, a small incision was made on the common carotid artery between the ligation and carotid bifurcation and a 0.26 mm monofilament nylon suture was inserted using forceps. After occlusion of the right middle cerebral artery for 2 h, the suture was withdrawn and the incision was stitched.

### Experimental treatments

According to previous studies (Shen et al. 2019a, 2019b), all rats were kept in an experimental barrier environment for one-week adaptive feeding. After one week of acclimatization, they were randomly assigned to five groups (*n* = 12): sham, model (MCAO/R), study treatment (PF + CG, 40 mg/kg + 20 mg/kg), LY294002 (20 mg/kg) and study treatment + LY294002 (PF + CG + LY294002, 40 mg/kg + 20 mg/kg + 20 mg/kg). After 24 h of MCAO/R, the sham and model groups were treated with oral saline (1 mL/100 g), the other three experimental groups were administered with affinity components or intraperitoneal injection of inhibitors for 7 consecutive days. Tissues were collected 2 h after the final drug or saline administration. The overall experimental period was one week.

### Neurologic deficits

The modified Neurological Severity Score (mNSS) was assessed 24 h after reperfusion, according to a previous study (Zhao et al. 2019). Mild injury was scored from 0 to 6 points, moderate injury from 7 to 12 points, and severe injury from 13 to 18 points.

### Cerebral infarction

The brain tissue was cut into thin slices, which were stained with 2% tetrazolium chloride and incubated at 37 °C for 30 min. They were observed with Image J software, and the volume of cerebral infarction was calculated using the following formula: (contralateral hemisphere volume – no infarct ipsilateral hemisphere volume)/contralateral hemisphere volume × 100.

### Brain edoema

The brain was quickly removed and weighed (wet weight). Then, it was dehydrated at 105 °C for 24 h and weighed again (dry weight). The percentage of cerebral edoema was calculated as following: (1 – dry weight/wet weight) × 100.

### Oxygen-glucose deprivation reperfusion (OGD/R) model

According to previous studies (Shen et al. 2019a, 2019b), mouse hippocampal HT22 cells were obtained from Shanghai Kanglang Biological Technology Co., Ltd. (Shanghai, China) and cultured in DMEM supplemented with 10% heat-inactivated foetal bovine serum (FBS), penicillin (100 μg/mL), and streptomycin (100 μg/mL). HT22 cells were passaged at least twice and then plated at 8 × 10^4^ cells/mL in DMEM supplemented with 10% FBS for 24 h. Then, cells were refreshed with glucose-free DMEM and placed in an anaerobic chamber (37 °C, 94% N_2_, 5% CO_2_) for 8 h. Subsequently, HT22 cells were reoxygenated in a 37 °C incubator with 5% CO_2_ for 6 h.

### Experimental cell groups

The cells were divided into five groups: control group, model group (OGD/R), study treatment group (PF + CG, 40 mg/mL + 20 mg/mL), inhibitor group (LY294002, 20 mg/mL), and study treatment + inhibitor group (PF + CG + LY294002, 40 mg/mL+ 20 mg/mL + 20 mg/mL). At the beginning of reoxygenation, the OGD/R group was exposed to normal medium. Three groups (PF + CG, LY294002 and PF + CG + LY294002) were exposed to drug-containing medium for 6 h. The control group was cultured in normal medium and incubated for 6 h.

### CCK-8, ROS, LDH, MDA, and SOD determination assay

Viability of cells was evaluated by CCK-8 assay, according a previous study (Jiang et al. 2019). ROS, LDH, MDA, and SOD release was detected with the corresponding kits (Jiancheng, Nanjing, China), according to the manufacturer's instructions.

### Cell apoptosis analysis

Five groups of cells were digested with EDTA-free trypsin, resuspended in 200 mL staining buffer and loaded with Annexin V-FITC/PI at 4 °C for 30 min. The apoptotic rate was detected by flow cytometry with FACSCanto II system (Becton Dickinson), while the percentage of apoptotic cells was calculated with FlowJo software.

### Western blot analysis

After treatment, the cells were harvested in a radio-immunoprecipitation assay (RIPA) lysis buffer containing a protease inhibitor cocktail (PMSF). After centrifugation at 10,000 rpm for 10 min at 4 °C, the supernatant was collected and the total protein concentration was measured using the bicinchoninic acid (BCA) protein assay. Proteins were separated by 10% SDS-PAGE and transferred onto PVDF membranes. The membranes were then incubated overnight at 4 °C in 5% bovine serum albumin (BSA) solution with the following antibodies: rabbit anti-PI3K (1:1000 dilution), anti-AKT (1:1000), anti-p-PI3K (1:1000), anti-p-AKT (1:1000), anti-Bcl-2 (1:1000), anti-BAX (1:1000), anti-GSK-3β (1:1000), and anti-β-actin (1:1000). After three washes with TBST, the membranes were incubated with biotinylated goat anti-rabbit IgG (H + L) (1:3000) for 1 h at room temperature. Chemiluminescence was analysed using ImageJ software.

### Statistical analysis

Data were expressed as mean ± standard deviation (SD). Differences between groups were analysed by one-way analysis of variance, and pairwise comparisons were performed using LSD test. A *p* value < 0.05 was considered statistically significant. The SPSS 19.0 software (IBM, NY, USA) was used for statistical analysis.

## Results

### PF + CG improved behavioural outcomes in MCAO/R rats

**Figure 2.**
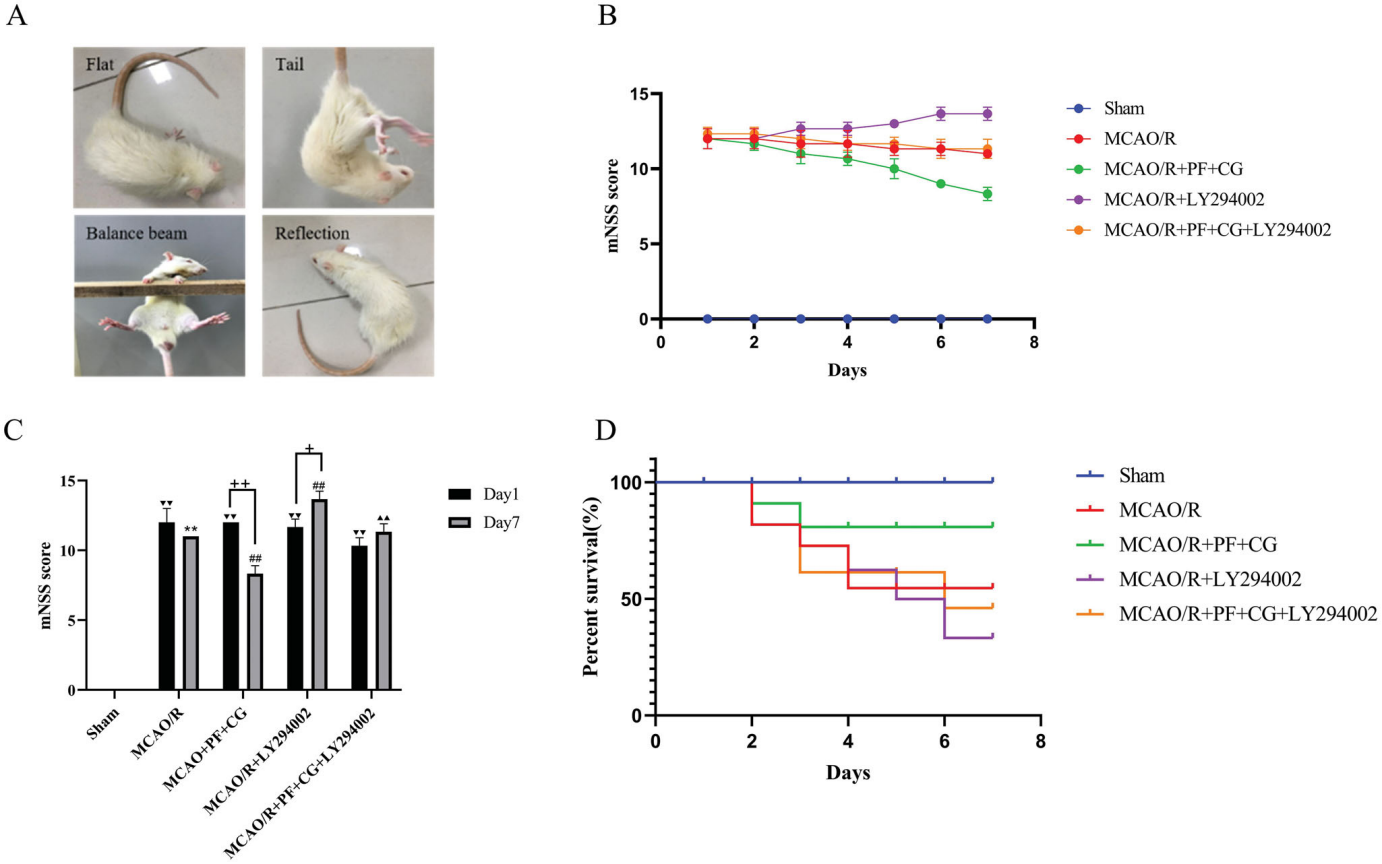
mNSS score results. (A) Schematic representation of neurological function. (B) Changes of neurological scores after seven days. (C) Statistical comparison of neurological scores between the 1st and the 7th day. (D) MCAO/R rats’ survival curve. Results are expressed as mean ± SD (*n* = 6) and are compared with the sham group. ^▼▼^ *p* < 0.01. ** *p* < 0.01 compared with the sham group on the 7th day. ^#^ *p* < 0.05. ^##^ *p* < 0.01 compared with the first day ^+^ *p* < 0.05. ^++^ *p* < 0.01 compared with theLY294002 group on the 7th day. ^▲▲^ *p* < 0.01.

After reperfusion for 24 h, a neurological assessment was performed in MCAO/R rats. Evaluations of exercise performance, sensory function, reflexes, and balance are presented in Figure 2(A). Score results are shown in Figure 2(B and C). On day 1, the mNSS score in the MCAO/R group (12.33 ± 0.82) was significantly higher than the sham group (*p* < 0.01). On day 7, the mNSS score was significantly higher in the MCAO/R group (11 ± 0) than the sham group (*p* < 0.01). The mNSS score was significantly lower in the study treatment group (8.67 ± 0.52) than the MCAO/R group (*p* < 0.01), whereas it was significantly increased in the LY294002 group (13.83 ± 0.41, *p* < 0.01). The mNSS score was significantly lower in the study treatment + LY294002 group (11.67 ± 0.52) than the LY294002 group (*p* < 0.01). Compared with day 1, the mNSS score was significantly lower in the study treatment group (8.67 ± 0.52, *p* < 0.01) and significantly higher in the LY294002 group (13.83 ± 0.41, *p* < 0.05) on day 7.

To further investigate the effects of PF + CG on the survival of rats subjected to MCAO/R, the number of surviving rats was recorded seven days after surgery. Results are displayed in a survival curve, as shown in Figure 2(D). The study treatment (PF + CG) improved the survival rate (83.33%), whereas LY294002 had an opposite effect (50%).

### PF + CG reduced cerebral infarct volume and brain edoema in MCAO/R rats

**Figure 3.**
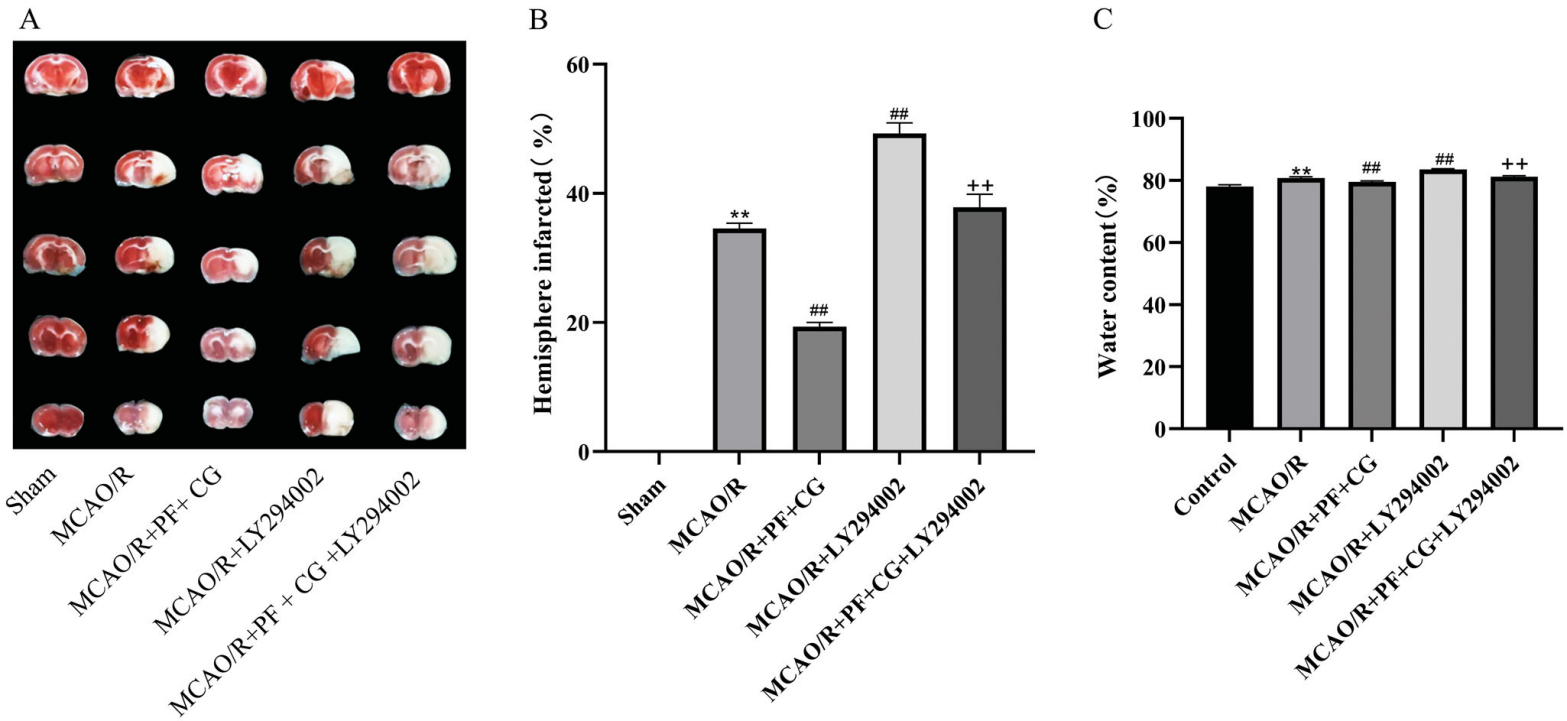
Effect of PF + CG on cerebral infarct volume and brain edoema in MCAO/R rats (A) TTC staining results. (B) Statistical results of cerebral infarction volume. (C) Statistical results of brain edoema. Results are expressed as mean ± SD and are compared with the sham group. *p < 0.05. ** *p* < 0.01 compared with the MCAO/R group. ^#^*p* < 0.05. ^##^*p* < 0.01 compared with the LY294002 group ^+^*p* < 0.05. ^++^*p* < 0.01.

Cerebral infarct volume was evaluated by TTC staining of brain sections. Representative TTC-stained brain sections are shown in Figure 3(A), with corresponding volume data are displayed in Figure 3(B). Cerebral infarct volumes were significantly increased in the MCAO/R and LY294002 groups (34. 44 ± 0.98, 49.35 ± 1.57, *p* < 0.01), whereas it was reduced by the study treatment (19.45 ± 0.96, *p* < 0.01). When compared with the LY294002 group, the study treatment + LY294002 significantly decreased the cerebral infarct volume (37.96 ± 2.22, *p* < 0.01).

Brain edoema results are shown in Figure 3(C). The brain edoema was significantly increased in the MCAO/R and LY294002 groups (80.82 ± 0.41, 83.56 ± 0.24, *p* < 0.01), while reduced by the study treatment (79.52 ± 0.23, *p* < 0.01). Compared with the LY294002 group, the study treatment + LY294002 significantly decreased the brain edoema (81.18 ± 0.35, *p* < 0.01).

### PF + CG significantly increased the levels of p-PI3K/PI3K, p-AKT/AKT and bcl-2/BAX, and reduced GSK-3β expression in MCAO/R rats

**Figure 4.**
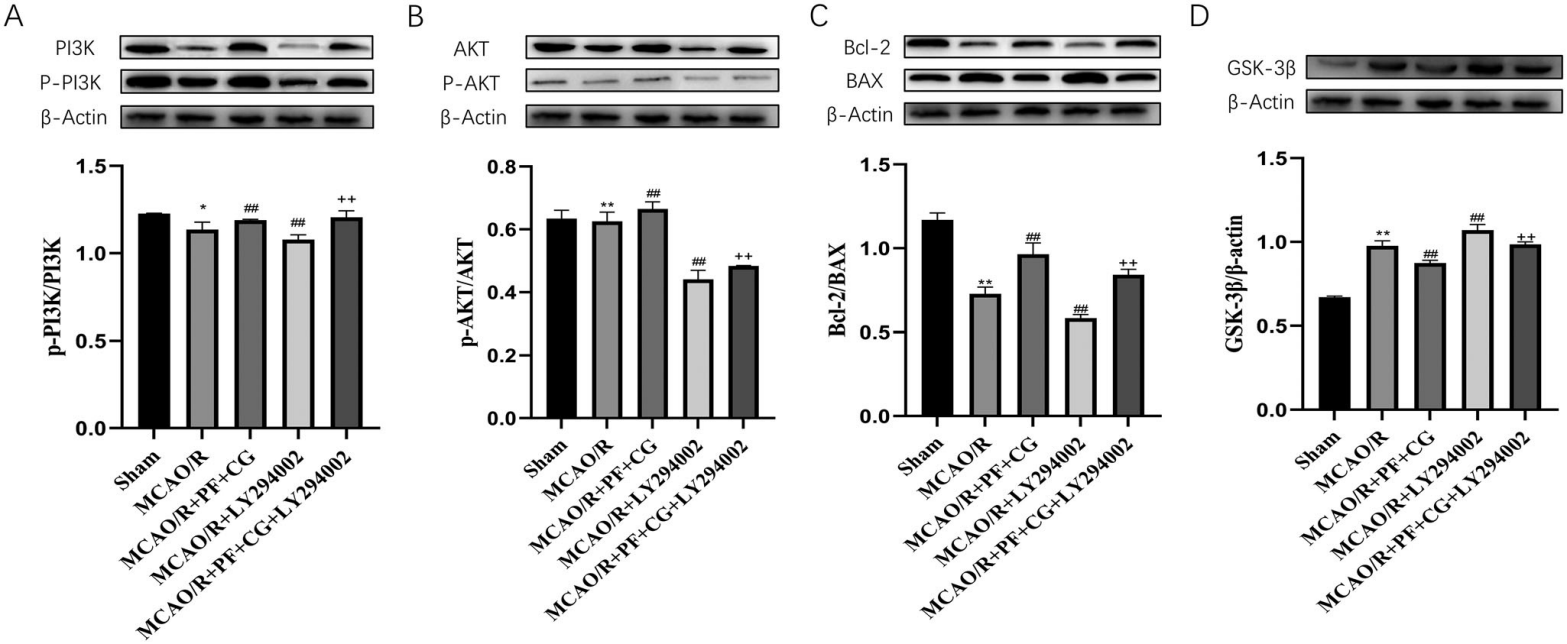
PI3K/p-PI3K, AKT/p-AKT, Bcl-2/BAX and GSK-3β/β-actin expression in MCAO/R rats. (A) Expression of PI3K, p-PI3K, β-actin measured with western blot and protein expression of p-PI3K/PI3K. (B) Expression of Akt, p-Akt, β-actin measured with western blot and protein expression of p-Akt/Akt. (C) Expression of Bax, Bcl-2, β-actin measured with western blot and protein expression of Bcl-2/Bax. (D) Expression of GSK-3β, β-actin measured with western blot and protein expression of GSK-3β/β-actin. Results are expressed as mean ± SD and are compared with the sham group. * *p* < 0.05. ** *p* < 0.01 compared with the MCAO/R group. ^#^*p* < 0.05. ^##^*p* < 0.01 compared with the LY294002 group. ^+^*p* < 0.05. ^++^*p* < 0.01.

The Western blot results showed that the study treatment significantly increased the level of p-PI3K/PI3K, p-AKT/AKT and Bcl-2/BAX, and reduced the expression of GSK-3βin ischaemic brains (*p* < 0.01) (Figure 4(A–D)). On the contrary, LY294002 significantly reduced the level of p-PI3K/PI3K, p-AKT/AKT and Bcl-2/BAX, and increased the expression of GSK-3β in ischaemic brains (*p* < 0.01). The study treatment + LY294002 significantly increased the expression of p-PI3K/PI3K, p-AKT/AKT and Bcl-2/BAX, and reduced the expression of GSK-3β in ischaemic brains compared with LY294002 only (*p* < 0.01).

### PF + CG increased the viability of OGD/R cells, elevated the levels of SOD and reduced the levels of ROS, LDH, and MDA

**Figure 5.**
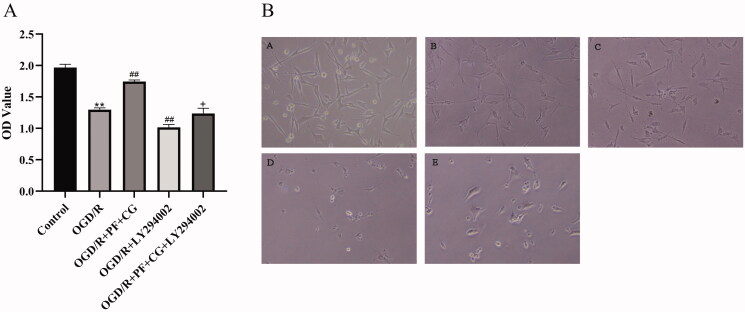
Effect of PF + CG on the viability of OGD/R cells. (A) CCK-8 measurement results. (B) Cell morphology of controls (A) Control (B) OGD/R (C) OGD/R + PG + CG (D) OGD/R + LY294002 (E) OGD/R + PF + CG + LY294002 groups. Results are expressed as mean ± SD and are compared with the sham group. *p < 0.05. **p < 0.01 compared with the MCAO/R group. ^#^*p* < 0.05. ^##^*p* < 0.01 compared with the LY294002 group. ^+^*p* < 0.05. ^++^*p* < 0.01.

The OGD/R injury significantly decreased the viability of HT22 cells compared with the control group (1.30 ± 0.03, *p* < 0.01). PF + CG enhanced the viability of HT22 cells (1.75 ± 0.02, *p* < 0.01), whereas LY294002 showed an opposite effect (1.02 ± 0.05, *p* < 0.01). PF + CG + LY294002 enhanced the viability of HT22 cells compared with LY294002 only (1.24 ± 0.08, *p* < 0.05) (Figure 5).

**Figure 6.**
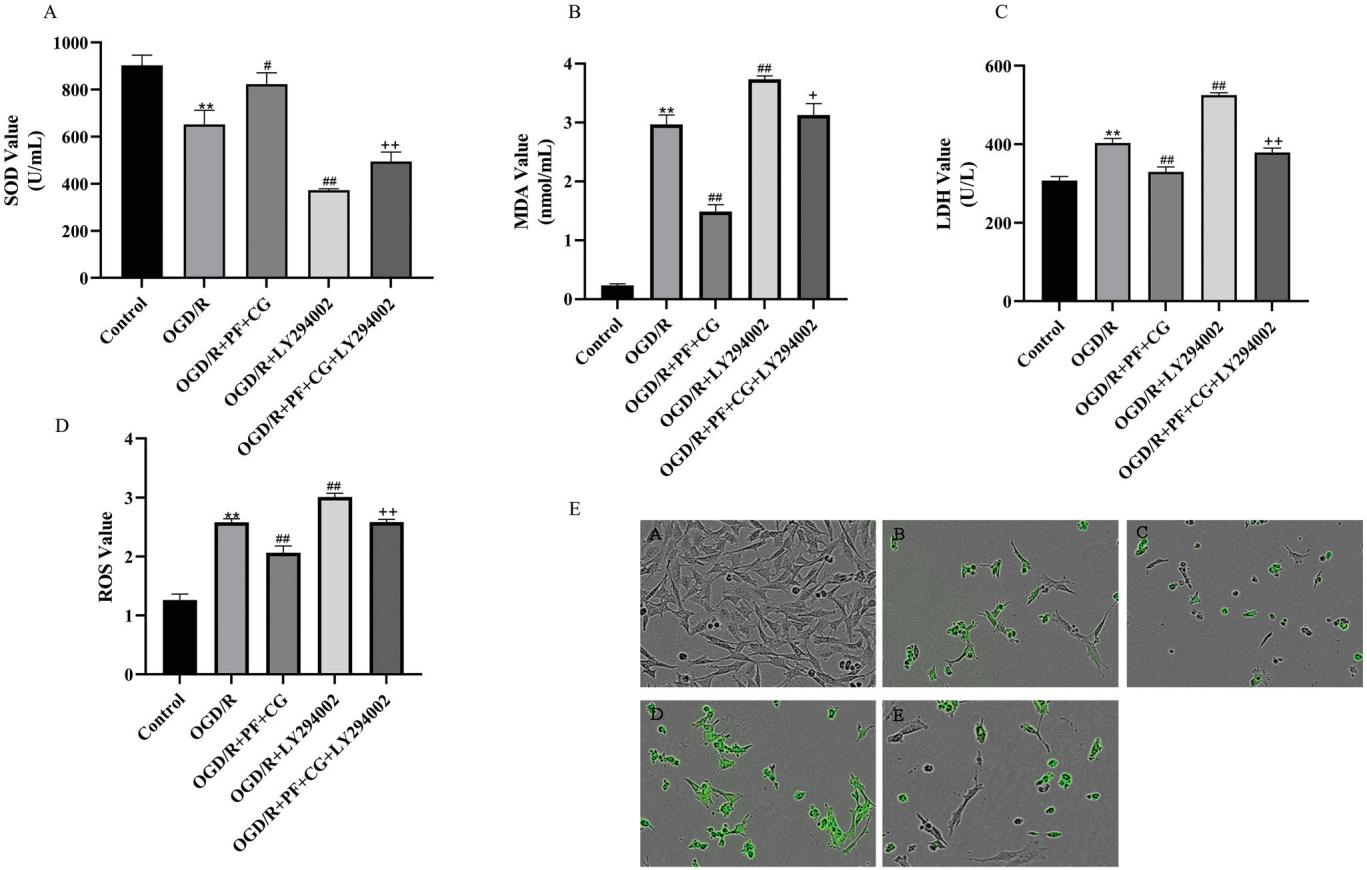
Effect of PF + CG on oxidative stress. (A) SOD. (B) MDA. (C) LDH. (D) ROS. (E) ROS cell fluorescence of controls (A) Control (B) OGD/R (C) OGD/R + PG + CG (D) OGD/R + LY294002 (E) OGD/R + PF + CG + LY294002. Results are expressed as mean ± SD and are compared with the sham group. *p < 0.05. **p < 0.01 compared with the MCAO/R group. ^#^*p* < 0.05. ^##^*p* < 0.01 compared with the LY294002 group. ^+^*p* < 0.05. ^++^*p* < 0.01.

Due to OGD/R injury, SOD levels were significantly decreased while ROS, LDH, and MDA levels were increased (2.58 ± 0.06, 403.37 ± 11.49, 2.97 ± 0.16, *p* < 0.01) (Figure 6). The study treatment significantly increased SOD level and decreased ROS, LDH and MDA expression (822.66 ± 48.41, 2.07 ± 0.11, 323.91 ± 12.34, 1.49 ± 0.12, *p* < 0.01). LY294002 significantly decreased SOD level and increased the level of ROS, LDH, and MDA (372.98 ± 6.04, 3.01 ± 0.06, 525.25 ± 6.06, 3.74 ± 0.06, *p* < 0.01). OGD/R＋PF + CG + LY294002 significantly increased SOD level and decreased ROS, LDH and MDA expression when compared with LY294002 only (494.99 ± 39.59, 2.59 ± 0.05, 379.12 ± 11.13, 3.13 ± 0.19, *p* < 0.05, *p* < 0.01).

### PF + CG had an anti-apoptotic effect on OGD/R cells

**Figure 7.**
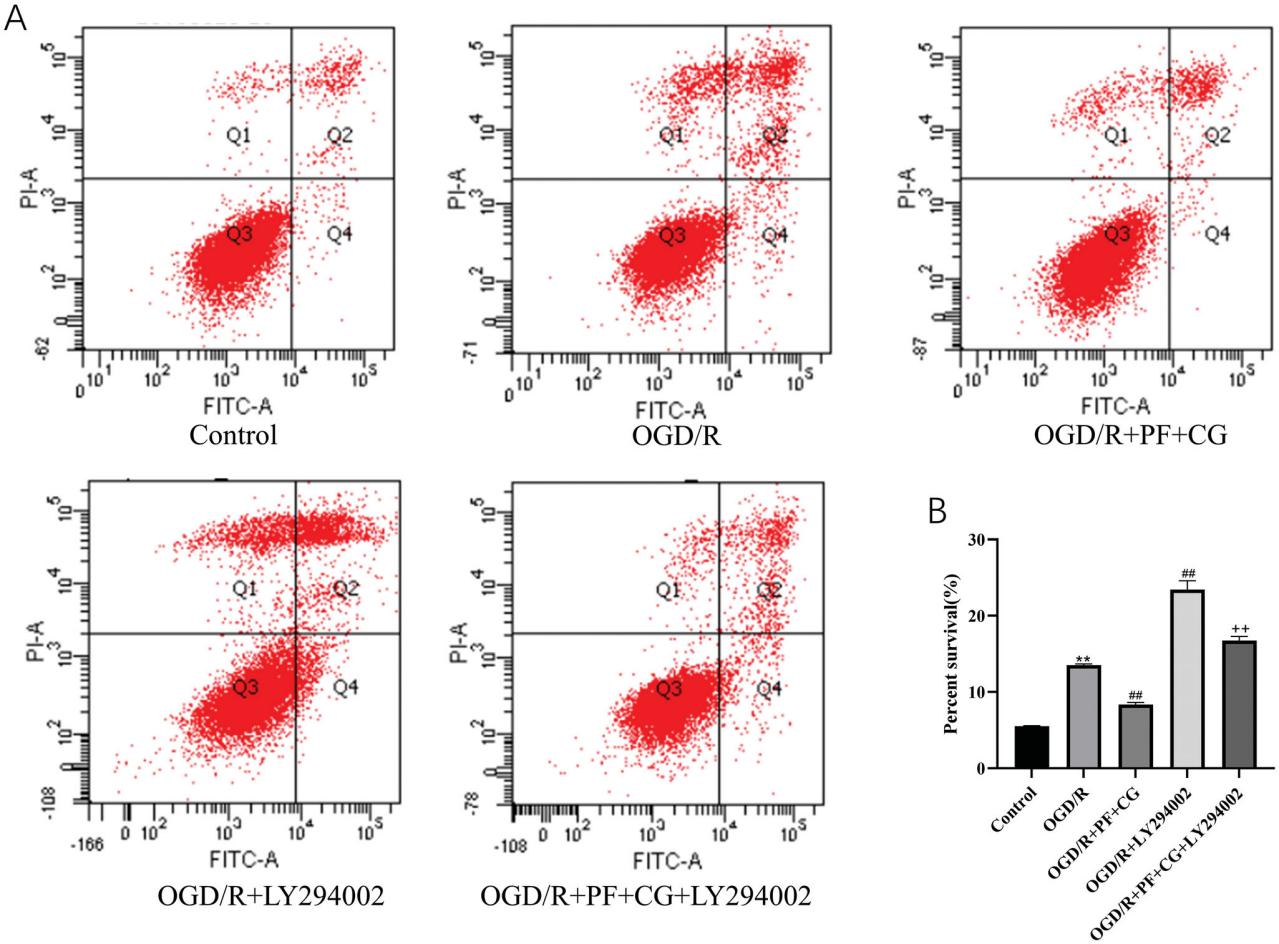
Effect of PF + CG on apoptosis. (A) Flow cytometry results. (B) Precent survival. Results are expressed as mean ± S.D. and are compared with the sham group. *p < 0.05. **p < 0.01 compared with the MCAO/R group. ^#^*p* < 0.05. ^##^*p* < 0.01 compared with the LY294002 group. ^+^*p* < 0.05. ^++^*p* < 0.01.

As shown in Figure 7, the OGD/R injury significantly increased the apoptotic rate of cells compared with the control group (*p* < 0.01). On the contrary, the study treatment significantly decreased apoptosis (*P* < 0.01). The LY294002 treatment significantly increased apoptosis when compared with the OGD/R group (*p* < 0.01), as the study treatment + LY294002 group when compared with LY294002 only (*p* < 0.01).

### PF + CG increased the levels of p-PI3K/PI3K, p-AKT/AKT and Bcl-2/BAX, and reduced GSK-3β expression in OGD/R cells

**Figure 8.**
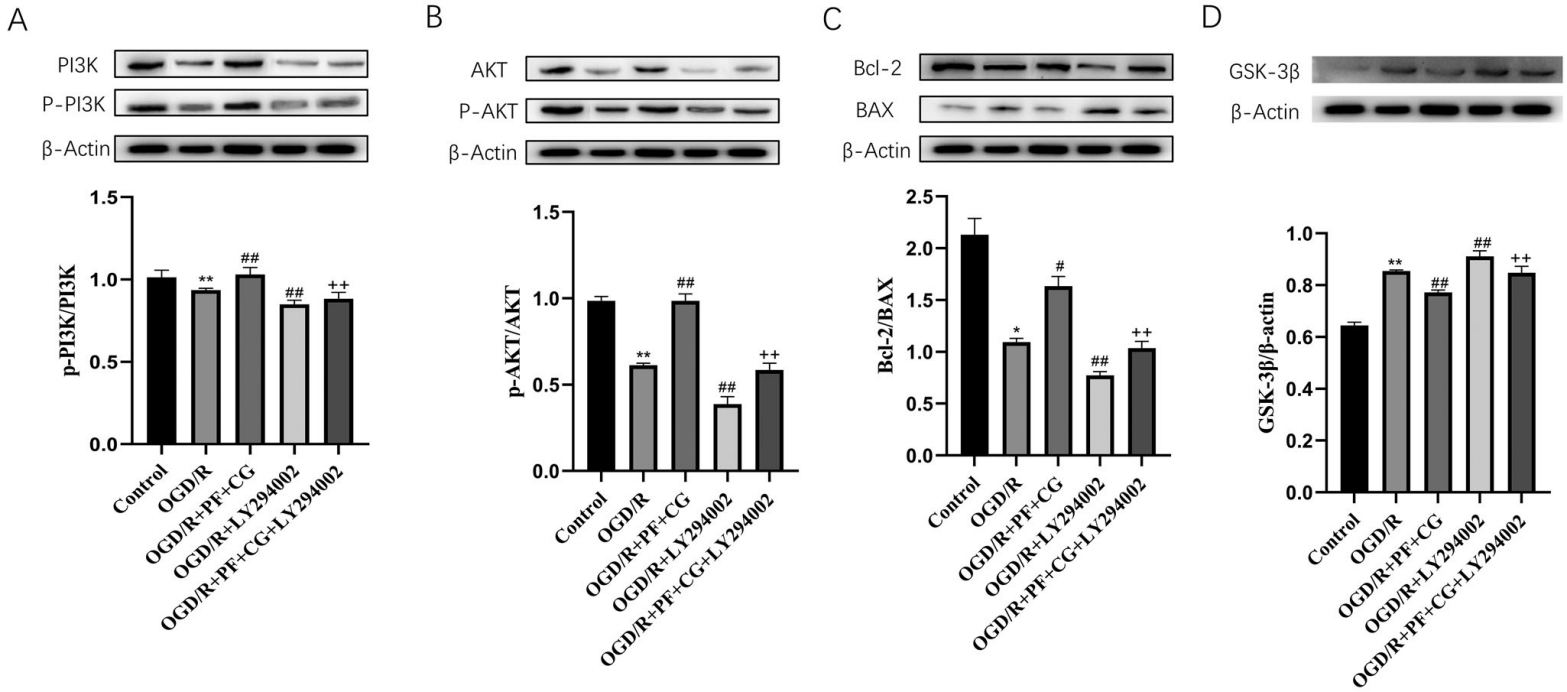
PI3K/p-PI3K, AKT/p-AKT, Bcl-2/BAX and GSK-3β/β-actin expression in MCAO/R rats. (A) Expression of PI3K, p-PI3K, β-actin measured with the western blot and protein expression of p-PI3K/PI3K. (B) Expression of Akt, p-Akt, β-actin measured with western blot and protein expression of p-Akt/Akt. (C) Expression of Bax, Bcl-2, β-actin measured with western blot and protein expression of Bcl-2/Bax. (D) Expression of GSK-3β, β-actin measured with western blot and protein expression of GSK-3β/β-actin. Results are expressed as mean ± S.D. and are compared with the sham group. * *p* < 0.05. ** *p* < 0.01 compared with the MCAO/R group. ^#^*p* < 0.05. ^##^*p* < 0.01 compared with the LY294002 group. ^+^*p* < 0.05. ^++^*p* < 0.01.

The western blot showed that the study treatment significantly increased the expression of p-PI3K/PI3K, p-AKT/AKT and Bcl-2/BAX, and reduced the expression of GSK-3β compared with the OGD/R group (*p* < 0.01) (Figure 8(A–D)). The LY294002 treatment significantly reduced the expression of p-PI3K/PI3K, p-AKT/AKT and Bcl-2/BAX and increased GSK-3β expression compared with the OGD/R group (*p* < 0.01). The study treatment + LY294002 significantly increased the level of p-PI3K/PI3K, p-AKT/AKT and Bcl-2/BAX, and reduced the expression of GSK-3β compared with LY294002 only (*p* < 0.01).

## Discussion

The pathogenesis of ischaemic stroke involves various mechanisms, among which oxidative stress damage and apoptosis appear to be the most critical events (Li et al. 2019). In our study, we investigated the protective effect of PF + CG in a MCAO/R rat model and OGD/R-induced neuronal apoptosis *in vitro*.

We showed that the administration of PF + CG decreased behavioural deficits, cerebral infarct volume and brain edoema in MCAO/R rats and increased cell viability after OGD/R treatment. In addition, the treated neurons exhibited increased levels of p-PI3K/PI3K, p-AKT/AKT and Bcl-2/BAX, and a reduced GSK-3β expression. These results indicate that PF + CG may have a synergistic protective effect on ischaemia/reperfusion injury.

The evaluation of neurobehavioral impairment has been studied by many methods in animals with various forms of ischaemic lesions. The Narrow-Alley Corner test was used to evaluate asymmetric motor dysfunction (Yonemori et al. 1998), while the sticky dot test was adopted as a complex measure of perception and motor coordination (Kadam et al. 2009). The Chimney test was applied to evaluate motor coordination before and after the MCAO-induced brain ischaemia (Nieoczym et al. 2019). In our study, the mNSS was assessed 24 h after reperfusion in accordance with previous studies (Singh et al. 2018a, 2020).

Oxidative stress plays an important role in the pathological process of ischaemic stroke and is critical for the development of neuronal damage. It is caused by reactive oxygen species (ROS) and is likely to lead to neuronal death and ultimately brain injury after reperfusion (Li et al. 2018; Mahalakshmi and Kurian 2018). In the process of cerebral ischaemia/reperfusion, considerable amounts of ROS are produced, aggravating oxidation stress injury. SOD is a scavenger of superoxide radicals and prevents oxidative damage of neuronal cells (Xu et al. 2018). During reperfusion, the membrane phospholipids are extremely sensitive to be attacked by ROS (Zhao et al. 2018), thereby producing lipid peroxidation, destroying the integrity of the cell membrane, and inducing apoptosis. In our study, PF + CG increased the SOD levels and reduced ROS, LDH, and MDA in OGD/R neuronal cells (Singh et al. 2018b).

The PI3K/AKT signalling pathway is involved in the regulation of neuronal apoptosis after hypoxic-ischaemic injury. AKT is a serine/threonine-specific protein kinase that synergizes with PI3K to promote proliferation and survival of cells (Song et al. 2019; Tong et al. 2020). Bcl-2 and BAX are homologous proteins with opposite effects in neuronal cells, with Bcl-2 serving to prolong cell survival and Bax acting as an accelerator of apoptosis (Badr et al. 2019). GSK-3β is localized in neurons and is a key regulator of cell homeostasis (Gao et al. 2020). During cerebral ischaemia/reperfusion, signals that originate from the extracellular environment can phosphorylate and activate PI3K. Activated PI3K regulates its downstream target protein through phosphorylation, including AKT. Activated AKT mediates further downstream responses, such as phosphorylation and inhibition of pro-apoptotic Bcl-2 and GSK-3β. Our results suggest that PF + CG may attenuate ischaemia/reperfusion and OGD/R injury.

Since we investigated the neuronal protective effects of PF + CG in MCAO/R rats and OGD/R cells, we further explored the impact of PF + CG on the PI3K/AKT signalling pathway. LY294002, a protein kinase that specifically inhibits the PI3K/AKT signalling pathway, aggravated neurobehavioral outcomes, cerebral infarct volume, and brain edoema in MCAO/R rats, whereas MCAO/R + PF + CG + LY294002 showed opposite effects. Furthermore, LY294002 reduced the viability of HT22 cells, decreased SOD, p-PI3K/PI3K, p-AKT/AKT, and Bcl-2/BAX expression and increased ROS, LDH, MDA and GSK-3β expression, whereas MCAO/R + PF + CG + LY294002 demonstrated opposite results. Altogether, the results suggest that PF + CG may attenuate ischaemia/reperfusion injury via the PI3K/AKT signalling pathway.

## Conclusions

PF + CG exhibited protective effects against cerebral ischaemia/reperfusion injury in MCAO/R rats and alleviated OGD/R injury in neuronal cells. The mechanism is attributed to the activation of the PI3K/AKT signalling pathway, providing a pharmacological rationale for the development of PF + CG as a treatment for IS.

## Data Availability

Data supporting the findings of this study are deposited in the Centre for Research Data repository (10.4121/uuid:4fa615c9-f5e9-4144-b678-173d2a13c560).
